# Transparent ZnO Thin-Film Deposition by Spray Pyrolysis for High-Performance Metal-Oxide Field-Effect Transistors

**DOI:** 10.3390/ma12203423

**Published:** 2019-10-19

**Authors:** Junhee Cho, Seongkwon Hwang, Doo-Hyun Ko, Seungjun Chung

**Affiliations:** 1 Electrical Engineering Division, Department of Engineering, University of Cambridge, CB3 0FA Cambridge, UK; jhcho@khu.ac.kr; 2 Department of Applied Chemistry, Kyung Hee University, Yongin, Gyeonggi 17104, Korea; dhko@khu.ac.kr; 3 Photo-Electronic Hybrids Research Center, Korea Institute of Science and Technology, Seoul 02792, Korea; sky27179@kist.re.kr

**Keywords:** metal oxide semiconductors, spray pyrolysis, thin film transistors, transparent oxide, zinc oxide

## Abstract

Solution-based metal oxide semiconductors (MOSs) have emerged, with their potential for low-cost and low-temperature processability preserving their intrinsic properties of high optical transparency and high carrier mobility. In particular, MOS field-effect transistors (FETs) using the spray pyrolysis technique have drawn huge attention with the electrical performances compatible with those of vacuum-based FETs. However, further intensive investigations are still desirable, associated with the processing optimization and operational instabilities when compared to other methodologies for depositing thin-film semiconductors. Here, we demonstrate high-performing transparent ZnO FETs using the spray pyrolysis technique, exhibiting a field-effect mobility of ~14.7 cm^2^ V^−1^ s^−1^, an on/off ratio of ~10^9^, and an *SS* of ~0.49 V/decade. We examine the optical and electrical characteristics of the prepared ZnO films formed by spray pyrolysis via various analysis techniques. The influence of spray process conditions was also studied for realizing high quality ZnO films. Furthermore, we measure and analyze time dependence of the threshold voltage (*V*_th_) shifts and their recovery behaviors under prolonged positive and negative gate bias, which were expected to be attributed to defect creation and charge trapping at or near the interface between channel and insulator, respectively.

## 1. Introduction

Field-effect transistors with transparent metal oxide semiconductors (MOSs) have emerged as a promising driving component in future display applications based on their intrinsic advantages over opaque silicon-based candidates. MOSs, in general, offer high carrier mobility, sufficient to drive recent organic light-emitting diode (OLED)-based displays [1]. Moreover, high optical transparency of MOSs based on the large band gap provides huge versatility in transparent electronic applications. In particular, to be useful in display applications, they should incorporate high switching speeds [2]. In this respect, MOSs stand on a more competitive position to silicon-based and organic semiconductors because it is hard for them to overcome either their intrinsic optical or performance limits.

MOSs also provide a wide range of processing versatility. From vacuum-based sputtering [3,4], atomic layer deposition (ALD) [5,6], and metal-organic chemical vapour deposition (MOCVD) [7,8] to solution processes of dip coating [9], spin-coating [10], ink-jet [11,12], and spray pyrolysis [13,14,15], a wide range of processing methods have been employed to fabricate MOSs. In particular, vacuum-based deposition techniques have restrictions in process versatility due to their high manufacturing costs and high process temperature. Solution process, on the other hand, has merits in its simplicity, low cost, and potential for low process temperature, which can satisfy the increasing need for flexible/plastic/bendable displays.

Despite their great potential, the electrical instability of MOS-based field-effect transistors (FETs) is one of the critical issues for next-generation FETs. The electrical stability is directly related to display performance and reliability. Especially in OLED displays driven by the drain current of FETs, it can be more critical as it leads to fluctuation in brightness of individual pixels, resulting in nonuniformity [16]. Regardless of semiconductors, a number of potential microscopic processes affecting stability and reliability have been generally identified, including charge trapping in the insulator, charge trapping at or near the interface between insulator and semiconductor, and a change in the defect distribution in the semiconductor. These vary the transfer characteristics of the FETs in the form of change in threshold voltage, sub-threshold slope (*SS*), field-effect mobility (µ_FE_), or a combination of these [17,18]. However, the dominant mechanism can be determined by the bias stress test and the intrinsic properties of the semiconductor and gate dielectric. Therefore, it is important to identify and characterize a number of interfacial traps [19,20,21].

In this paper, we report high-performance ZnO FETs using the spray pyrolysis technique. Vaporized ZnO precursor solution is delivered in the form of mist to a heated substrate where the desired films are formed via the chemical reaction. Spray pyrolysis offers better electrical performance of MOS-based FETs because the high substrate temperature during the film deposition gives rise to rapid evaporation of impinging liquid droplets, leading to the increase of film growth [22,23]. By adapting the spray pyrolysis method, we could achieve MOS-based thin-film devices on large-area platforms maintaining excellent material properties. Nontoxic and optically transparent ZnO film was obtained in polycrystalline forms via the spray pyrolysis technique and later realized as FETs. Through process optimization in spray distance, pressure, and fabrication temperature, the ZnO FETs using spray pyrolysis present outstanding electrical characteristics of high mobility (~14.7 cm^2^ V^−1^ s^−1^). The FETs also presented high-quality switching properties, showing an on/off ratio of ~10^9^ and *SS* of ~0.49 V/decade. Furthermore, the electrical instabilities of our ZnO FETs, which are induced by positive and negative gate bias stress, are examined and discussed, and the resulting, apparently distinctive, instability tendencies of the positive and negative bias stress are explained as identifying dominant mechanisms, defect creation, and charge trapping in positive and negative bias, respectively.

## 2. Materials and Fabrication

### 2.1. Fabrication of Zinc Oxide FETs via Spray Pyrolysis

**Figure 1:**
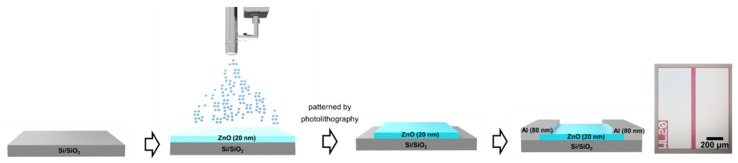
Fabrication procedure of ZnO field-effect transistors (FETs) by spray pyrolysis deposition and an optical image of the channel region. The ZnO (purple) channel layer and Al *S*/*D* electrode (light grey) were sequentially deposited on the Si/SiO_2_ substrate (dark grey) via spray pyrolysis and thermal evaporation, respectively. The channel region was patterned by photolithography to minimize the leakage current.

Figure 1 shows the procedure of fabricating ZnO FETs by spray pyrolysis. In preparing the precursor for the ZnO deposition, a 0.1 M solution of zinc acetate dihydrate (Zn(CH_3_CO)_2_·2H_2_O) (Sigma Aldrich, St. Louis, MS, USA, 99.0%) was dissolved in methanol under vigorous stirring conditions for one hour at room temperature. The standard oxygen plasma treatment was performed on the cleaned Si/SiO_x_ substrate with a duration time of 400 s to eliminate undesirable organic residue and increase surface energy as well, which results in improving the wetting properties of the methanol-based ZnO precursor. Then, the substrate was kept on a hot plate at 400 °C during the entire spray process. ZnO thin films were fabricated using the spray pyrolysis method. Aerosols of the prepared ZnO precursor solution were deposited onto the heated substrate with a distance of 15 cm from the nozzle via a conventional hand-gun airbrush, which is described in Figure 1. All the spraying processes were performed in a fume hood, and compressed air with controlled pressure was used to deliver aerosols to the substrate. The spray pyrolysis process was performed with the sequence of 15 s spray and 45 s rest at the pressure of 15 psi until obtaining a 20 nm-thick semiconducting layer. Then, the channel layer was defined by photolithography wet etching in 0.0625 M hydrochloric acid (HCl). Aluminium (Al) source and drain (*S*/*D*) electrodes (80 nm) were deposited using a thermal evaporator and then patterned by a lift-off process through standard photolithography to achieve ZnO FETs with channel width (*W*)/channel length (*L*) = 1000 μm/50 μm.

#### 2.1.1. Patterning the ZnO Semiconducting Layer via Wet Etching

The etching process for sprayed ZnO semiconductor film was accomplished as follows: AZ5412E, a positive photoresistor (PR), was spin-coated on the spray-processed ZnO film and then soft-baked at 100 °C on a hotplate for a minute. After being exposed for 7 s to UV light via an EVG 620 photo aligner, the film was developed in an AZ 351 developer for 15 s and hard-baked at 120 °C for two minutes. After the photolithography process, the ~20 nm-thick ZnO film was etched in diluted HCl (0.0625 mM) for around 2 min and consequently cleaned in distilled water (DI water). After drying, the PR, finally, was removed through a cleaning process of acetone, isopropyl alcohol (IPA), and DI water in sequence. A 3D optical profiler (Wyko NT1100, Billerica, MA, USA) was used to confirm whether the channel layer was well-defined. 

#### 2.1.2. Deposition and Patterning of Al S/D Electrodes

The patterned S/D electrodes of ZnO FETs were formed via a lift-off process as follows: The same AZ 5412E, which was used as a positive PR for patterning ZnO semiconductor film, was employed here as a negative PR because it was reversible. The PR was spin-coated onto the patterned ZnO film. After soft baking on a hotplate at 100 °C for 1 min, UV light exposure was followed using an EVG aligner for 9 s. Then, we could obtain the image reverse by using a special crosslinking agent in the photoresistor, via activation by inversion baking at 110 °C for 2 min on a hotplate. After a flood exposure and developing in AZ 351B for 10–15 s, the positive PR areas were dissolved and the cross-linked areas remained. Then, the device was cleaned by DI water and blow-dried in nitrogen before depositing an 80 nm-thick Al electrode layer using thermal evaporator, Edwards E306A. Consequently, the PR was removed by cleaning in acetone, IPA, and DI water in sequence, and the well-patterned S/D electrodes were achieved.

### 2.2. Characterization

Transmittance spectra were examined by a Unicam UV2 UV–Vis Spectrometer (Cambridge, UK). X-ray diffraction (XRD) patterns and Atomic Force Microscope (AFM) images were measured using a Bruker X-ray Diffraction D8 X-ray diffractometer (Billerica, MA, USA, 40 kV and 40 mA) and Bruker MultiMode 8 Scanning Probe Microscope from NanoScope^®^ V controller (Billerica, MA, USA), respectively. A Renishaw micro Raman spectrometer (Wotton-under-Edge, Gloucestershire, UK) with a 325 nm He-Cd laser and 40× objective lens (NA 0.5) was employed for photoluminescence analysis. The laser power and grating used were 0.5 mW and 2400 grooves/mm, respectively.

Electrical characteristics and retention measurement of the FETs were collected a by Tektronix Keithley 4200 semiconductor characterization system (SCS) (Beaverton, OR, USA) in a dark box at room temperature. The linear field effect mobility was extracted from the slope of transfer curve in the linear regime using Equation (1) [13,24].

(1) $$\mu_{e,lin}\;=\;\frac{L}{C_{i}W}\frac{\partial I_{D}}{\partial V_{G}}$$

where *L* and *W* are the length and width of the semiconductor channel, respectively, and *C_i_* is the geometrical capacitance of the dielectric layer.

The *SS* value, the inverse of the maximum slope of the transfer characteristics, was extracted using Equation (2) [24].

(2) $$SS\;=\;\left(\frac{\partial V_{G}}{\partial log_{10}\left(I_{D}\right)}\right)$$

To investigate the instability behavior of our ZnO FETs, the prolonged constant gate voltage of ±20 V were applied for 20 ks for positive and negative bias stress, respectively. For better interpretation, the spray-pyrolysized ZnO FETs were annealed at 120 °C for 1 h to equilibrate the defect density in the ZnO channel layer before electrical bias stress. During the stress test, the gate-to-source voltage (*V_G_*) was applied as maintaining 0.1 V of drain voltage (*V_D_*) for a specific period of time. Then, the linear regime transfer characteristics were measured. This process was repeated up to 20 ks for both the positive and negative bias stress test. In analysing the instability behaviours of the ZnO FETs, we focused on the electrical transfer characteristics in the linear region, which applied a low *V_D_* to minimize the effect of drain voltage on the electric field across the dielectric. During recovery, all terminals were left floating in ambient air at room temperature.

## 3. Results and Discussions

### 3.1. Characterization of Spray-Pyrolysized ZnO Thin Films

The ZnO films deposited by spray pyrolysis were formed as the mists of vaporized precursors were transformed and reacted on the surface of the heated substrate. Because all the individual precursors behaved together for the reaction, patterning and interface control was critical to achieve lower *off*-state currents [23]. Therefore, we patterned the active layer via photolithography (Figure 1) and performed oxygen plasma surface treatment on the SiO_2_ gate dielectric in order to improve the performance of the ZnO FETs. The patterning of channel layer was expected to lead a high *on*/*off* ratio as it significantly lowered the drain current in *off*-states compared to the previously reported results (Table 1). Additionally, from the surface treatment, we could improve the wetting properties of the methanol-based ZnO precursor as it eliminated undesirable organic residue and increased surface energy. Through the process of optimization, we could conclude that the overall performance of our spray-pyrolysized ZnO FETs was competitive with the previously reported ZnO-based FETs using a spray pyrolysis method.

**Figure 2:**
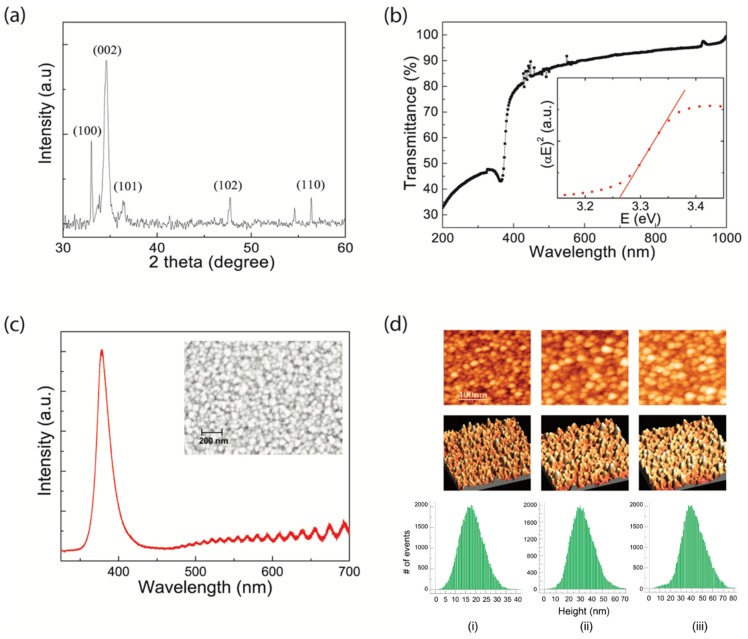
The properties of ZnO films formed by spray pyrolysis technique under optimized conditions (15 psi, 25 cm, and 400 °C of spray pressure, distance, and substrate temperature, respectively). (**a**) X-ray diffraction spectrum and (**b**) optical transmission (%) spectra of the ZnO film. The inset shows the optical absorption coefficient (α) versus photon energy (E) characteristics. The horizontal intercept of the line indicates 3.26 eV as the film’s optical band gap. Both X-ray diffraction (XRD) and optical transmittance spectrum are from the ZnO film itself as the ones from the substrate are subtracted. (**c**) Photoluminescence (PL) spectra and SEM (inset) image of the film. (**d**) Atomic Force Microscope (AFM) images (phase and topography) with distribution of surface roughness of ZnO films sprayed at various spray pressure conditions: (**i**) 15 psi, (**ii**) 30 psi; and (**iii**) 45 psi.

The quality of the spray-coated ZnO thin films was evaluated by analyzing crystallinity and surface roughness via X-ray diffraction (XRD) and an atomic force microscope (AFM). Figure 2a shows the diffraction patterns of ZnO film sprayed on a Si/SiO_x_ substrate at 400 °C. For easier analysis, the background diffraction patterns of the Si/SiO_x_ substrate were subtracted. The XRD pattern presents a preferred orientation of (002) with the significantly highest peak and secondary peaks of (100), (101), (102), and (110), which indicate that our spray process produced polycrystalline ZnO films with a hexagonal wurtzite phase. The result is also consistent with earlier reports that undoped ZnO films by spray pyrolysis technique were highly oriented along the (002) direction [25,26]. We also extracted the optical bandgap throughout the UV–Vis spectrum measurement in order to additionally qualify the ZnO thin films. Figure 2b shows the UV–Vis transmission spectra of sprayed ZnO films on quartz. For the optical characterization, the direct optical band gap was calculated from Tauc plots from the transmittance measurement using the following equation [27]:

(3) $$\alpha \hbar \omega =B{\left(\hbar \omega -E_{g}\right)}^{2}$$

where α, ℏω, and *E_g_* indicate absorption coefficient, photon energy (*E*), and optical bandgap, respectively. From a fit of the plot of (*αE*)^2^ vs. the photon energy (*E*) (the inset in Figure 2b), we obtained the value of 3.26 eV. This value is consistent with that of polycrystalline ZnO, ~3.3 eV, and of other spray-processed ZnO films [28,29].

To analyse the electronic structure of ZnO thin films, we additionally adapted the photoluminescence (PL) spectroscopy, as it could provide information that would enable us to identify surface, interface and impurity levels, and interface roughness [30]. Figure 2c presents the PL spectra of the spray-coated ZnO films of various thickness. Sharp emissions in the UV region (~380 nm) and broad emission bands in the visible region (~520 nm) are observed. Although there is artificial interference with the PL signal in the range of 480 to 700 nm by Raman edge filter, the intensity of the visible emission is undoubtedly low when compared with the UV emission. The emission in the UV region was expected to be associated with the band gap emission. Meanwhile, the visible emission involves the transition of an electron from the conduction band (or a shallow level close to the band) to a trap level approximately 2 eV below the conduction band edge. When considering that the intensity of the visible emission band directly corresponds to the level of defect states [31,32], we can assume that our spray-coated ZnO films had a low concentration of defects.

The surface morphologies of optimized ZnO films (15 psi, 25 cm, and 400 °C of spray pressure, distance, and substrate temperature, respectively) were also investigated from SEM (inset of Figure 2c) and AFM images ((i) in Figure 2d). The measured root–mean–square (RMS) roughness (500 nm × 500 nm) was 3.3 nm. The image, additionally, shows that grain sizes were distributed from 30 nm to 40 nm. The SEM image indicates the similar topology of the ZnO films compared to their AFM images.

### 3.2. The General Electrical Characteristics of Spray-Pyrolysized ZnO FETs

**Figure 3:**
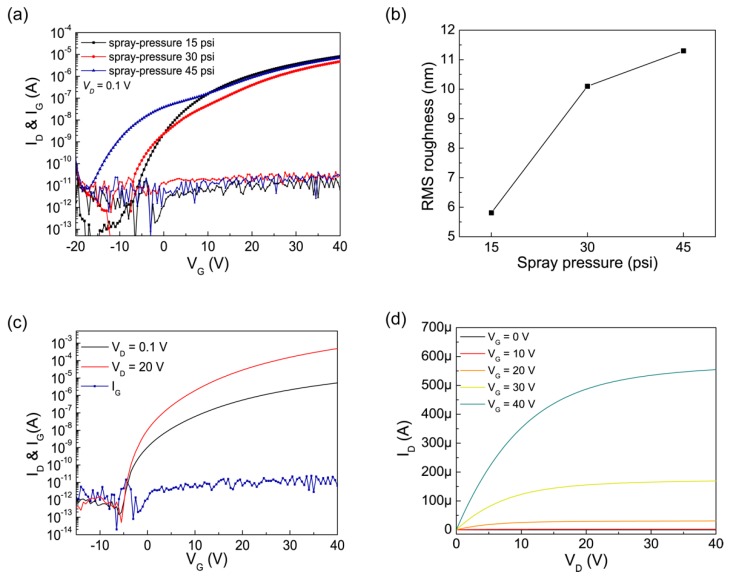
(**a**) Drain and gate current vs. *V*_G_ characteristics of ZnO FETs, fabricated using various spray pressures (15 psi, 30 psi, 45 psi) in log scale (*V_D_* = 0.1 V). (**b**) Root–mean–square (RMS) roughness of ZnO thin films formed using three different spray pressures: 15 psi, 30 psi, 45 psi. (**c**) Transfer characteristics (log(*I_D_*) – *V_G_*) of the staggered bottom-gate ZnO FETs using spray pyrolysis technique at linear (at *V_D_* = 0.1 V) and saturation (at *V_D_* = 20 V) regions with the gate leakage (*I_G_*). (**d**) Output characteristics (*I_D_* – *V_D_*) of the ZnO FETs.

The spray-coated ZnO FETs were achieved through the process of optimization by tuning fabrication conditions, including spray pressure, the distance from the nozzle to the substrate, and process temperature. Among those, we especially focused on the spray pressure as it presented a critical influence on the FET properties and was directly related to the deposition rate, whilst not relatively well established when compared with other elements such as temperature [15,28]. Figure 3a presents the transfer characteristics of the ZnO FETs fabricated using various spray pressures of 15 psi, 30 psi, and 45 psi at a spray distance of 25 cm.

Table 2 summarizes the key parameters of the device performance as a function of spray pressure. Overall, the FETs fabricated at a low pressure of 15 psi provided better electrical performance, higher field-effect mobility, lower *SS*, and higher drain on-current and lower off-current. In particular, the *SS* showed the most significant variation. The *SS* of the ZnO FETs formed at pressures of 30 psi and 45 psi showed some fluctuation and the level of the fluctuation became higher as the spray pressure increased, whereas smooth transfer characteristics were observed in the FETs at a pressure of 15 psi with high densification. This result is consistent with the previous report that the deposition pressure in ZnO-based FETs could be strongly associated with densification [34]. Figure 2d and Figure 3b show the AFM images with the detailed distributions of surface roughness of each film and root–mean–square (RMS) roughness of the ZnO films as a function of spray pressures (15, 30, and 45 psi, respectively). The lower spray pressure was adapted, and the lower surface roughness was achieved, which is concomitant with an increase in densification led by low spray-pressure deposition.

*SS* indicates the total trap density (*N_t_*) involving the bulk trap density (*N_bulk_*) of the semiconductor itself and the interface trap density (*D*_e_) at or near the interface between the semiconductor and gate dielectric material, using the following equation: [35]

(4) Nt = Nbulk + Dit = [{SS log10(e)(kBTq)}−1](Ciq)

where *k_B_* is Boltzmann’s constant, *T* is absolute temperature, and *C_i_* is the capacitance of the gate insulator. This formula points out that the better *SS* value in low spray pressure can be directly related to the reduction of the total trap density. It is hard to identify whether *N_bulk_* and/or *D_it_* contributed to the improvement of *SS*. However, based on the report that the densification was strongly affected by *N_bulk_* rather than *D_it_*, [34] we can assume that the reduction of *N_bulk_* by increased densification played the dominant role to improve the *SS* value. Moreover, when considering that the field-effect mobility and *SS* were related to the density of shallow traps near the conduction band and the deep-level traps, respectively [36], the better field-effect mobility and *SS* for the FETs using low spray pressure indicate that the high densification led to an overall improvement in trap density.

Figure 3c,d shows the transfer and output characteristics of the optimized spray-pyrolysized ZnO FETs (spray pressure of 15 psi, spray distance of 25 cm, and substrate temperature of 400 °C) in linear and saturation modes. In measuring the transfer characteristics, the gate-to-source voltage (*V_G_*) was swept in steps of 0.5 V from −20 V to +40 V, while drain-to-source voltage (*V_D_*) was 0.1 V and 20 V for linear and saturation regions, respectively. The field effect mobility, the drain current on/off ratio, and threshold voltage (*V*_th_) were ~14.7 cm^2^ V^−1^ s^−1^, ~10^9^, and ~3.5V, respectively. These parameters are important for FETs because field effect mobility determines the current drive of the device, and for switching application, values greater than 10^6^ of the on/off ratio are required for devices in conventional display applications [37]. The FETs also presented the subthreshold swing (SS) of ~0.49 V/decade, which describes the necessary *V*_G_ to increase I_D_ by one decade. SS is the feature that indicates how efficiently the device can be turned on [38]. Because all the individual elements of precursors react together on the surface of the hearted substrate to form a film, it is hard to control the channel layer, and thus, patterning is essential in the spray pyrolysis process [23]. For this reason, we patterned the active layer via photolithography (Figure 1), and as a result, improved the performance of the ZnO FETs, especially in switching properties. From patterning the channel layer, we could drop our off-state current from ~10^−8^ to ~10^−13^ A, which lead to good on/off ratio characteristics of 10^9^ and also improved the characteristics of SS from ~4 to ~0.49 V/decade. The gate leakage sustained in the low level also points out that the channel layer was well controlled during the transistor operation (Figure 3c). We also examined output characteristics of the ZnO FETs. As seen in Figure 2d, we could obtain the clean output curve with no current crowding observed, which indicated the Ohmic contacts formation.

Through the optimization, we could obtain the overall performance of our spray-pyrolysized ZnO FETs, unlike the previously reported ZnO-based FETs using the spray pyrolysis method (Table 1). Particularly, several merits were superior to the others, having more advantages in switching device application, such as the display backplane, SS ~0.49 V/decade, *V*_th_ ~3.5 V, and on/off ratio >10^6^ when applying a relatively small volume of V_D_, 0.1 V.

### 3.3. The Instability Behaviours of Spray-Pyrolysized ZnO FETs

Typically, the electrical instabilities of ZnO-based FETs have resulted in two proposed mechanisms. The first is carrier trapping at or near the channel/insulator interfaces. The second mechanism is the defect creation in the channel or at the channel/insulator interfaces that increase the density of deep-gap states. Since the distinction between the two mechanisms in amorphous silicon-based TFTs was experimentally reported, the state creation and the charge trapping have been studied as dominant mechanisms of electrical instability behaviors of TFTs and they have been determined by the degree of changes in *SS* [39]. Many research groups have studied sub-gap density of states (DOS) modeling, device simulation, thermal activation energy analysis, and the sub-gap state extraction based on electrical/optical experiments, but the mechanisms are not clearly defined yet [40,41,42]. To investigate the electrical instability of our ZnO FETs fabricated by spray pyrolysis, we analyzed their bias stress-induced stretched exponential time dependence, changes of transfer characteristics including *SS* and hysteresis, and their recovery behaviors of spray-processed ZnO FETs under electrical (both positive and negative) bias stress.

#### 3.3.1. The Electrical Instabilities of Spray-Pyrolysized ZnO FETs under Positive Bias Stress

**Figure 4:**
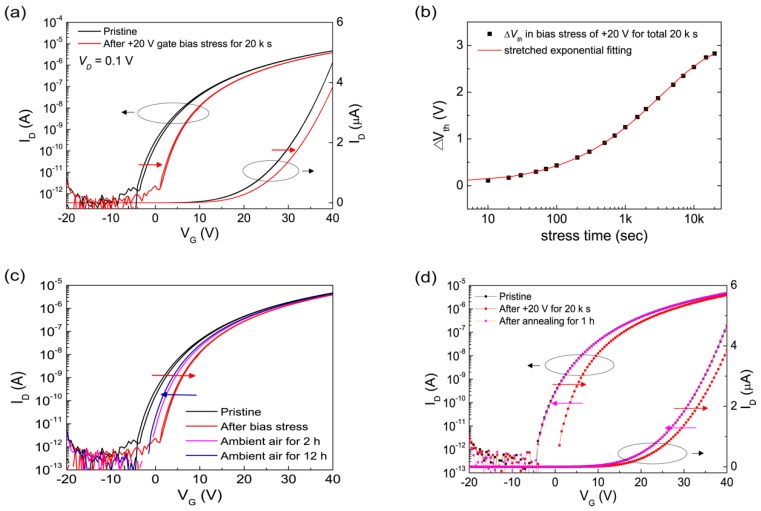
(**a**) The instability behaviours of transfer characteristics (log(*I_D_*) – *V_G_* and *I_D_* – *V_G_*) of the ZnO FETs (at V_D_ = 0.1 V) under prolonged positive (+20 V) gate bias for 20,000 s at room temperature (R.T.). The transfer curve shifts parallel to positive direction. (**b**) *V*_th_ shifts as a function of stress time in positive (+20 V) bias stress (PBS) at R.T. (**c**) The recovery behaviour of ZnO FETs at R.T in ambient air after PBS. (**d**) The shifts of the transfer characteristics under thermal annealing (at 120 °C) in ambient air.

Figure 4a presents the shifts of transfer curves as a function of time before and after positive bias stress (+20 V) for 20 ks. The shifted transfer curve in parallel indicates that the threshold voltage shift ($\Delta V_{th}$) occurred with minimal change in *SS*. In both transfer characteristics before and after electrical bias stress, a hysteresis loop with minimal shift was observed. The threshold shift induced by positive gate bias stress followed stretched exponential behaviors, which is described by Equations (5) and (6) [43].

(5) ΔVth = ΔVtho[1−exp{−(tτ)β}]

(6) $$\tau ={\nu_{c}}^{-1}exp\left(\frac{E_{A}}{kT}\right)$$

where *τ* is the time constant that exhibits an activated behaviour, and *β* is the exponential stretch parameter. *ν_c_* and *E_A_* indicate the speed of light and the activation energy, respectively. This tendency has been known to be closely related with defect creation and, in case of ZnO-based FETs’ oxygen vacancy, expected to be one of the strong candidates [44,45,46,47]. Oxygen vacancy (*Vo*) is widely believed to be a donor and defects in the (uncharged) V_o_ state tend to be formed under electron accumulation [44]. However, based on density functional calculations, V_o_ is suggested as a deep donor rather than a shallow one, despite the fact that it has the lowest formation energy among the defects that act as donors [48,49,50]. In addition, there was a small degradation (10%) in the field-effect mobility observed during the positive bias stress test. This is speculated to be predominantly related to the intrinsic defects of the ZnO channel layer, but further investigations are needed to fully understand the behaviour.

The recovery behaviours of the spray-processed ZnO FETs supported our argument. The positive bias stress (PBS)-induced transfer characteristics of the FETs were recovered to a certain level in ambient air as they shifted toward the prestressed one, but after a period of 12 h, they were rather far from fully recovering, as seen in Figure 4c. On the other hand, the annealing process led the device back to the prestress condition. With annealing at 120 °C for 1 h in air, the transfer characteristics of the FETs were fully recovered. As presented in Figure 4d, further annealing did not affect the electrical characteristics of the ZnO FETs. This indicates that the thermal annealing only played the role of removing defects that had been created during PBS. These results imply that the positive gate bias-induced threshold voltage shifts as a function of stress time are induced by defects created in the deep level, and thus require thermal energy to be recovered.

#### 3.3.2. The Electrical Instabilities of Spray-Pyrolysized ZnO FETs under Negative Bias Stress

**Figure 5:**
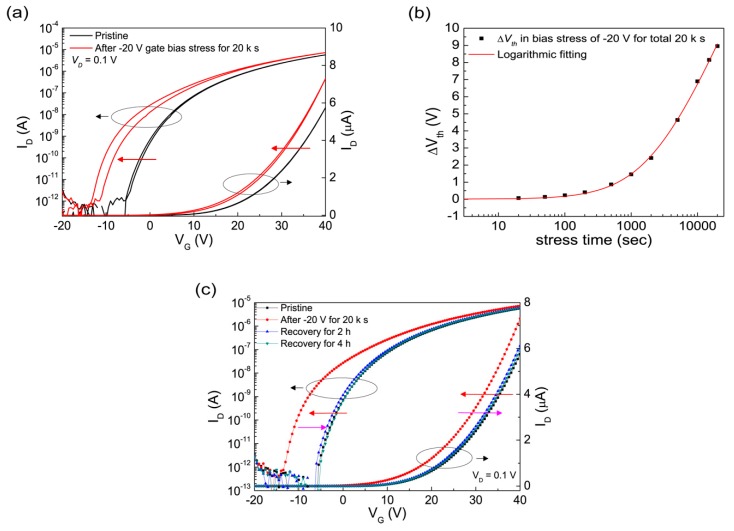
(**a**) The instability behaviours of transfer characteristics (log(*I_D_*) – *V_G_* and *I_D_* – *V_G_*) of the ZnO FETs (at *V_D_* = 0.1 V) under prolonged negative (−20 V) gate bias for 20 ks at R.T. The transfer curve shifts in parallel to negative direction. (**b**) *V*_th_ sfhit as a function of stress time in negative bias stress (NBS) at R.T. (**c**) Recovery behaviour of ZnO FETs at R.T in ambient air after NBS.

In negative electrical gate bias, the electrical characteristics of the spray-processed ZnO FETs showed different tendencies when compared to the PBS ones. Figure 5a shows the threshold voltage shift properties of the ZnO FETs induced by negative bias stress (NBS). Unlike the PBS-induced *V*_th_ shifts following the stretched exponential fitting, the *V*_th_ shifts on NBS show A logarithmic dependence (Equation (7)), which comes along with little change in SS [51].

(7) $$\Delta V_{th}\;=\;r_{d}\;\log\left(1+\frac{t}{\tau }\right)$$

where *r_d_* is a decay rate constant, and *τ* is the time constant (Equation (6)).

These characteristics generally indicate that electron trapping at or near the channel/insulator interface without the creation of new defects was the main mechanism of the instability behaviors [52]. Furthermore, the widened hysteresis characteristic after NBS of −20 V gate bias for 20 ks indicates that the charge trapping with fast re-emission times (or fast state) was dominantly associated with the NBS instability behaviours [39]. In recovery, NBS induced the different characteristics when compared with PBS. While PBS requires thermal annealing, the shifted transfer characteristics under NBS were fully recoverable without additional supporting energy. After releasing at room temperature for 4 h, the transfer characteristics returned to the pre-stress ones; *V*_th_ shifted back; and hysteresis also became narrowed back to the pre-stress level. No further shifting was observed in the longer resting period at room temperature, which indicates that no further electrical behaviors were involved, except when releasing the trapped charges. Such bias-induced *V*_th_ shifts and release behaviors of the ZnO FETs under NBS point out that the charging trapping in a fast state could have been a main mechanism for NBS, whose energy to release the trapped interface charge was very low. The instability behaviors under NBS are consistent with the previous studies on ZnO TFTs: Deposited by radio frequency (RF) magnetron sputtering; logarithmic *V*_th_ shift with no effect on SS characteristics; and recovered at room temperature (R.T.). without any additional process, where the dominant mechanism was estimated to be the charge trapping at or near the interface of channel/insulator layers [19,51]. Although the slightly increased field-effect mobility was also observed, similar to the behavior under the positive bias stress, it was not a significant change when compared with the clearly changed *V*_th_ and recovery. As aforementioned, more studies are needed to clearly explain how the prolonged gate bias affects the mobility of ZnO FETs.

## 4. Conclusions

Stable and high-performance ZnO FETs were fabricated using a spray pyrolysis technique in an ambient atmosphere. Polycrystalline film ZnO was obtained via optimization of the spray pyrolysis process, the nature of which resulted in high field-effect mobility. Patterning using conventional photolithography enabled sharp switching properties with a high on/off ratio and a good *SS* characteristic. We examined the optical, morphological, and electrical properties of the FETs. Furthermore, we analyzed their gate bias-induced instabilities under prolonged positive (+20 V) and negative (−20 V) gate voltage for 20 ks. From the threshold voltage shifting with the bias stress and following recovery behaviors, we reached the conclusion that defect creation and charge trapping at or near the interface between semiconductor and gate insulator play a dominant role in electrical instability behaviors by positive and negative gate bias, respectively. As there are only a few studies on instabilities of ZnO-based FETs fabricated by spray pyrolysis, our approaches to investigate electrical stabilities can be considered a significant step to address commercialization of the MOS FETs based on the spray pyrolysis process.

## Figures and Tables

**Table 1:** Comparisons of the electrical characteristics of previously reported metal-oxide FETs by spray pyrolysis deposition.

Reference	Active Layer (Process Temperature)	Gate Dielectric	S/D Electrodes	Mobility (cm^2^ V^−1^ s^−1^)	On/Off	SS (V dec^−1^)
This work	ZnO (400 °C)	Thermally grown SiO_2_	Al	~14.7	10^9^	0.49
Adamopoulos et al. [13]	Li-ZnO (400 °C)	spray-pyrolysized ZrO_2_	Al	~85	10^6^	-
Afouxenidis et al. [14]	ZnO (400 °C)	spray-pyrolysized Al_2x−1_Ti_x_O_y_	Al	~10	10^6^	0.55
Bashier et al. [15]	ZnO (400 °C)	Thermally grown SiO_2_	Al	~15	10^6^	2.9
Wang et al. [33]	IZO (350 °C)	Thermally grown SiO_2_	Al	~4.5	10^6^	-

**Table 2:** The morphological and electrical characteristics of ZnO FETs formed at various spray pressures of 15 psi, 30 psi, and 45 psi.

Spray Pressure (psi)	Surface Roughness (nm in RMS)	Field-Effect Mobility (cm^2^ V^−1^ s^−1^)	*V*_th_ (V)	Sunthreshold Swing (V/Decade)	I_D_^ON^ (A)	I_D_^OFF^ (A)
15	5.81	14.7	3.6	0.78	3.9 × 10^−7^	~5 × 10^−13^
30	10.1	13.2	1.2	1.0	2.8 × 10^−7^	~7 × 10^−13^
45	11.3	9.9	−7.3	2.6	1.9 × 10^−7^	~1 × 10^−11^
